# Coverage, Adherence and Costs of Intermittent Preventive Treatment of Malaria in Children Employing Different Delivery Strategies in Jasikan, Ghana

**DOI:** 10.1371/journal.pone.0024871

**Published:** 2011-11-03

**Authors:** Edith Patouillard, Lesong Conteh, Jayne Webster, Margaret Kweku, Daniel Chandramohan, Brian Greenwood

**Affiliations:** 1 Department of Global Health and Development, London School of Hygiene and Tropical Medicine, London, England; 2 Institute of Global Health Innovation, Imperial College London, London, England; 3 Ghana Health Service, University of Ghana, Accra, Ghana; Universidade Federal de Minas Gerais, Brazil

## Abstract

**Background:**

Intermittent preventive treatment of malaria in children (IPTc) involves the administration of a course of anti-malarial drugs at specified time intervals to children at risk of malaria regardless of whether or not they are known to be infected. IPTc provides a high level of protection against uncomplicated and severe malaria, with monthly sulphadoxine-pyrimethamine plus amodiaquine (SP&AQ) and sulphadoxine-pyrimethamine plus piperaquine being the most efficacious regimens. A key challenge is the identification of a cost-effective delivery strategy.

**Methods:**

A community randomized trial was undertaken in Jasikan district, Ghana to assess IPTc effectiveness and costs using SP&AQ delivered in three different ways. Twelve villages were randomly selected to receive IPTc from village health workers (VHWs) or facility-based nurses working at health centres' outpatient departments (OPD) or EPI outreach clinics. Children aged 3 to 59 months-old received one IPT course (three doses) in May, June, September and October. Effectiveness was measured in terms of children covered and adherent to a course and delivery costs were calculated in financial and economic terms using an ingredient approach from the provider perspective.

**Results:**

The economic cost per child receiving at least the first dose of all 4 courses was US$4.58 when IPTc was delivered by VHWs, US$4.93 by OPD nurses and US$ 5.65 by EPI nurses. The unit economic cost of receiving all 3 doses of all 4 courses was US$7.56 and US$8.51 when IPTc was delivered by VHWs or facility-based nurses respectively. The main cost driver for the VHW delivery was supervision, reflecting resources used for travelling to more remote communities rather than more intense supervision, and for OPD and EPI delivery, it was the opportunity cost of the time spent by nurses in dispensing IPTc.

**Conclusions:**

VHWs achieve higher IPTc coverage and adherence at lower costs than facility-based nurses in Jasikan district, Ghana.

**Trial Registration:**

ClinicalTrials.gov NCT00119132.

## Introduction

Intermittent preventive treatment of malaria is the administration of a full course of an anti-malarial treatment to a population at risk at specified time points, regardless of whether or not they are known to be infected. The World Health Organization recommends IPT for the prevention of malaria in pregnant women (IPTp) [1] and infants (IPTi) [2] and the potential role of IPT as a malaria control strategy in children in areas of seasonal transmission risk is gaining increasing interest. A recent systematic literature review was conducted to assess the safety and efficacy of IPTc administered to children under five years of age in countries of the Sahel and sub-Sahel where malaria transmission is highly seasonal. Twelve studies were identified and a pooled analysis indicated that IPTc administered monthly offered a protective efficacy of 82% against clinical attacks and suggested an impact on all-cause mortality [3]. Different drug combinations were used in different settings with monthly sulphadoxine-pyrimethamine (SP) plus amodiaquine (AQ) and SP plus piperaquine being the most efficacious regimens. IPTc was safe with no serious drug related adverse events [3].

A key challenge for IPT is the identification of an appropriate delivery strategy. IPT is dispensed to pregnant women during their regular visits to antenatal clinics and to infants at routine EPI (Expanded Programme on Immunisation) visits. However, it is not clear how to reach children under five years of age who do not make regular contacts with routine health care services. Different delivery strategies have therefore been explored in different countries. Children received IPT from village health workers (VHWs) in Senegal and Ghana [4], [5], supplemented in the latter by health facility-based staff (including nurses working at outpatient departments (OPD) and nurses running EPI outreach clinics (EPI)) [5], or as in The Gambia, by mobile reproductive and child health trekking (RCH) teams [6]. When community and facility-based delivery strategies were compared, higher coverage rates were achieved when IPTc was dispensed by VHWs compared to nurses. During a randomised trial of IPTc using SP+AQ given over 3 months in The Gambia, 74% of children who received IPT from VHWs received at least the first dose of the 3 courses compared to 48% of children who received treatment from the RCH teams [6]. In another randomized trial of IPTc using SP+AQ given over 4 months in Ghana, 91% of children in the VHW arm of the trial received the first dose of at least 3 courses compared with 87% of children in the OPD+EPI arm [5]. From a policy maker's perspective, the choice of which delivery strategies to use for IPTc must take into account the resources required for implementing IPT through the different delivery options and the potential savings of using a particular strategy or combination of strategies. However, to date, there is limited evidence on which to base such a decision, with a single comparative cost analysis conducted during the Gambian study, which showed that IPTc was less costly when dispensed by VHWs than by RCH teams, at US$ 1.63 and US$ 3.47 per child covered respectively [6]. The study presented in this paper provides new information on the costs of delivering IPTc through different routes by drawing on the findings of a community randomised trial conducted in Ghana, during which IPTc was dispensed to children by OPD or EPI nurses or by VHWs [5].

## Methods

### Ethics Statement

The study was approved by the ethics committees of the London School of Hygiene and Tropical Medicine and of the Ghana Health Service/Ministry of Health.

### Study population

Both the community randomised trial and the costing study were carried out in Jasikan district, Volta region, Ghana. A detailed description of the district is given elsewhere [5]. Briefly, in 2004, the district population was estimated at 122,265 inhabitants, with most residents being subsistence farmers cultivating primarily rice and maize. The population was served by 2 hospitals, 9 health centres, 17 reproductive and child health clinics, 3 private maternity homes, 2 private clinics and an additional 80 outreach clinics providing EPI services. EPI coverage was high in the district, with around 80% of children aged one year and above fully vaccinated. In Ghana, malaria transmission is seasonal and occurs during the rainy season (April to November), with peaks from May to June and September to October. At the time of the study, insecticide treated nets (ITNs) were subsidized at public health centres and EPI outreach clinics, and vouchers to be used in private shops were available at antenatal clinics. However, only one third of households reported that they owned at least one bed net, of which 9.5% were ITNs. In Jasikan, children experienced between 3 to 6 clinical attacks annually, with severe malaria being the most common cause of hospital admission and death in children under five years of age. In 2006, artesunate and amodiaquine combination therapy became the recommended first line treatment for uncomplicated *Plasmodium falciparum* malaria in Ghana.

### Study design

The community randomized trial was designed to assess the effectiveness of IPTc in terms of coverage and adherence achieved through OPD, EPI and VHW delivery. Informed written consent was obtained from all the care takers of the eligible children before enrolment in the study.

The design of the trial has been reported in details elsewhere, including sample size calculations and sampling procedures [5]. Twelve villages were randomly selected to receive IPTc through OPD (3 villages), EPI outreach clinics (3 villages) or VHWs (6 villages). SP+AQ were dispensed to parents/care-takers of children aged 3–59 month-old in May, June, September and October 2006. Nine hundred and sixty-four children were enrolled, of whom 248 were in the OPD arm, 244 in the EPI arm and 472 in the VHW arm.

Children in each arm of the trial were similar in regard to age, gender, anthropometric indices, malaria parasite prevalence, household insecticide treated net ownership and whether they slept under a net the night before enrolment. Before the start of the IPTc intervention, nurses and VHWs received one day of training on how to identify drugs packed for each child, administer drugs and refer children to public health facilities.

At the beginning of May, June, September and October, IPTc drugs were delivered to health facilities and VHWs by the district health management team (DHMT) staff who participated in the trial. OPD nurses dispensed IPT to the study children during their facility's normal opening hours on two scheduled consecutive days and EPI nurses delivered IPTc during the course of their routine activities at one-day monthly outreach clinics. VHWs dispensed IPTc during three consecutive scheduled days from a central point of each village. Nurses and VHWs administered the first IPT dose, with tablets being crushed and mixed with sweetened water, whilst the two subsequent doses were administered at home by parents/care takers [5]. Drugs not distributed during the scheduled IPTc days were collected from dispensers a few days later by the same DHMT team that delivered the drug supplies. Visits to supervise nurses and VHWs during the administration of the first IPT dose and to monitor children's adherence to the second and third doses - five days after the administration of the first dose - were also conducted by DHMT team members.

### Coverage and adherence outcomes

IPTc effectiveness was measured for each delivery strategy (VHWs, OPD, EPI) and for the combined two strategies that used the routine public health system (OPD and EPI). Effectiveness was defined in terms of the number of children scheduled to receive IPTc who received treatment (coverage) and the number of doses taken by the covered children (adherence). Children were considered “fully” covered if they received the first supervised dose of all 4 courses, and “acceptably” covered if they received the first supervised dose of at least 3 courses. Coverage rates were calculated for IPTc delivered through each delivery strategy (Table 1). Adherence rates were measured by DHMT supervisors who visited a different sub-sample of children each month, with all children visited once during the study, and who asked parents/ care-takers about any IPT drugs left and whether the child had been sick [5]. The adherence rate was calculated by multiplying the proportion of children enrolled who were covered at that particular month by the proportion of children visited each month who took all three doses (Table 2). The proportion of children enrolled who took all three doses of four courses (defined as the proportion of children “fully covered and fully adherent”) was obtained by multiplying the proportions of children covered who took all three doses in May, June, September and October. The proportion of children who took all three doses of at least three of the four courses (defined as the proportion of children “acceptably covered and fully adherent”) was obtained by multiplying the proportions of children covered who took all three doses in at least three of the four months during which IPTc was dispensed. Adherence rates were calculated for children covered through the VHW delivery strategy or the facility-based strategy (OPD and EPI) (Table 2).

**Table 1:** IPTc coverage outcomes by delivery strategy.

	IPTc delivery strategies			
	Community-based delivery	Facility-based delivery		
	Village Health Workers (VHWs)*	OPD delivery (OPD)	EPI outreach delivery (EPI)	OPD+EPI delivery (OPD+EPI)*
Children enrolled in the study (%)	472 (100%)	248 (100%)	244 (100%)	492 (100%)
Children who received the first supervised dose of all 4 courses (“fully covered”)	326 (69.1%)	171 (69.0%)	151 (61.9%)	322 (65.4%)
Children who received the first supervised dose of 3 courses (“acceptably covered”)	116 (24.6%)	57 (23.0%)	58 (23.8%)	115 (23.4%)
Children who received the first supervised dose of 2 courses	25 (5.3%)	16 (6.4%)	27 (11.0%)	43 (8.7%)
Children who received the first supervised dose of 1 course	5 (1.0%)	4 (1.6%)	8 (3.3%)	12 (2.5%)
Children who did not receive the first supervised dose of any course	0 (0.0%)	0 (0.0%)	0 (0.0%)	0 (0.0%)

*These figures are slightly different from those published in the effectiveness paper [5] following recalculations.

**Table 2:** IPTc coverage and adherence outcomes achieved for each course by delivery strategy.

	Coverage, % of children who received at least the first dose* of each course		Adherence, % of children visited each month who took all 3 doses* [6]		Full adherence and coverage, % of children covered who took all 3 doses*	
	Community-based delivery (VHWs)	Facility-based delivery (OPD+EPI)	Community-based delivery (VHWs)	Facility-based delivery (OPD+EPI)	Community-based delivery (VHWs)	Facility-based delivery (OPD+EPI)
**June (course 1)**	100.0%	100.0%	92.3%	88.2%	92.3%	88.2%
**July (course 2)**	98.9%	97.5%	88.9%	86.9%	87.9%	84.8%
**September (course 3)**	93.6%	88.8%	84.9%	95.6%	79.5%	84.9%
**October (course 4)**	69.1%	65.4%	100.0%	97.5%	69.1%	63.8%

*A dose refers to the drugs taken each day of the 3-day IPTc drug regimen; dose 1 given on day 1 was supervised by nurses or VHWs and doses on day 2 and day 3 were the responsibility of parents/care-takers.

### Costing

Total unit financial and economic costs of IPTc delivered through the three different strategies were calculated from the perspective of the provider only. Household costs associated with accessing IPTc were not collected during this study. Cost data were collected following an ingredient approach [7] using records and activity data supplied by the DHMT. Care was taken to exclude resources related to research activities (e.g. costs of collecting the IPTc drugs that had not been dispensed at the end of each of the trial months). Resources were categorised under IPTc drug costs, training costs, delivery to distribution point costs, distribution to parents/care-takers costs, supervision costs and communications costs. Financial costs were actual expenditures incurred by the DHMT, including IPTc drugs, training allowances, personnel incentives, transport and supplies.

Drug costs were derived from the MSH drug indicator guide using the median buyer price for 2006 [8]. In order to account for drug wastage, the cost of a full tablet was calculated across all age groups, although some children had received half a tablet according to the age dependent dosage regimen. One monthly IPTc course was valued at US$0.05, with l tablet of SP at US$0.02 and a 3-day regimen of AQ at US$0.03.

During the one-day training course, VHWs, nurses and supervisors each received an attendance fee of US$5.00 supplemented by an allowance of around US$3.50 for refreshments and lunch.

During the four-month implementation period, a monthly incentive of US$10.00 was given to each VHW and nurse involved in IPTc distribution. The US$10.00 amount was set on the basis of incentives paid to VHWs and nurses involved in a previous IPTc study and in relation to payments made in other similar situations in Ghana at the time of the study [4]. Incentives were paid to two nurses generally working together at each clinic. Two DHMT supervisors received a monthly salary supplement of US$50.00 and one driver a monthly supplement of US$15.00. These incentives were equivalent to approximately 14% of the monthly salary received by DHMT supervisors and 6% of that received by a government driver.

Transport costs for attending IPTc training at the DHMT office were calculated using trainees' bus fare receipts. Transport costs for delivering IPTc to the distribution points and for conducting supervision were calculated using data on kilometres travelled, fuel consumption, fuel cost and vehicle maintenance costs collected from the project's vehicle logbook. Transport capital costs were calculated by dividing the vehicle replacement value at the time of the study by its useful life, estimated at 7.5 years, apportioned on the time that it was used for each activity under each delivery strategy. Communications costs were attributed equally between the different delivery strategies.

Economic costs represent the opportunity cost, or value of all resources used irrespective of whether these involve an additional direct cost. In this study, examples included the time spent by DHMT staff and nurses who already received a salary but who spent time on IPTc rather than on their usual duties and the time spent by VHWs who could otherwise have undertaken paid activities. Parents/care takers of children in the facility-based arms of the trial could pick-up their children's monthly IPT course during two pre-scheduled week days at their health centre's OPD or during the one day EPI outreach clinic. Based on the principal investigator's field experience and observations made during the trial, OPD and EPI nurses were estimated to spend 20% of their work day on IPTc. VHWs dispensed IPTc drugs at a central point in the village on three IPTc days and IPTc was assumed to be their only activity on these days. The time spent by nurses during training and when dispensing IPTc was valued using a senior midwife's gross salary including government allowances [7] (data obtained from the Ministry of Health's 2006 payroll records). The time spent by VHWs on IPTc-related work was valued using the 2005 minimum subsistence monthly wage in Ghana, inflated by 10% to reflect a plausible annual increase in 2006 (US$1.85) based on previous years' increases. The time spent by DHMT staff preparing and conducting training (a total of 2 days), distributing IPTc to the dispensing sites and for supervision was valued on the basis of a senior nurse's salary (including government allowances). The time spent by a driver delivering IPTc drugs and for supervision was valued on the basis of a government driver's salary (including government allowances).

Overheads, such as resources consumed for storing IPTc drugs (e.g. building, utilities) were not collected during the study. Costing of overhead resources used data available from a separate IPTc study conducted the same year in a neighbouring district, which had identified the rental value per square meter of hospital space used by the IPTc intervention to represent the opportunity cost of using that space [4]. Overhead costs were assumed for the study population over the six months of the intervention. Specifically, the value of overhead resources used was assigned on the assumption that the OPD delivery strategy used 8 square meters of storage space whilst the EPI based delivery strategy was assumed to use 80% of this space (because of the shorter storage period taking place at the facility under this delivery strategy). As for the community-based delivery strategy, it was estimated to use half the value of the storage resources used in the OPD delivery because IPTc drugs were stored in the homes of the VHWs and it was assumed that the opportunity cost of such space was lower than that at the health facility.

Finally, the vehicle capital cost was annualised using a discount rate of 3%. All costs were converted to US$ 2008 using an average exchange rate of US$1.00 = GHC9,781 (http://www.oanda.com/currency/average).

A sensitivity analysis was conducted on resources whose valuation was uncertain and likely to affect the cost differential between delivery strategies focusing on IPTc drug costs, discount rate for capital costs, share of time spent by nurses and VHWs on IPTc, amount of incentives received by VHWs and nurses, and training intensity.

## Results

### Total costs of IPTc delivery

Table 3 shows the total financial and economic costs of delivering IPTc through either VHWs or a health facility-based strategy. The total costs of IPTc delivered through OPD or EPI clinics are presented separately in Table 4.

**Table 3:** IPTc total financial and economic costs comparing community- and facility-based strategies.

Delivery strategies	Community-based delivery (VHWs)				Facility-based delivery (EPI+OPD)			
Number of children enrolled	472				492			
Costs	Total Financial (US$)	Cost Profile (%)	Total Economic (US$)	Cost Profile (%)	Total Financial (US$)	Cost Profile (%)	Total Economic (US$)	Cost Profile (%)
**IPTc drugs**	96	9%	96	6%	100	8%	100	6%
**Training**								
•Personnel	66	6%	146	10%	66	5%	154	9%
•Transport	13	1%	13	1%	10	1%	10	1%
**Delivering drugs to distribution points**								
•Personnel	15	1%	67	4%	30	3%	74	4%
•Transport	288	27%	264	18%	180	15%	166	10%
**Dispensing IPTc to parents/care-takers**								
•Personnel	240	23%	364	24%	480	39%	649	38%
•Transport	0	0%	0	0%	0	0%	0	0%
•Overheads	0	0%	29	2%	0	0%	53	3%
**Supervision of IPTc dispensing**								
•Personnel	80	8%	272	18%	91	7%	227	14%
•Transport	241	20%	202	14%	233	19%	221	13%
**Communications**	41	4%	41	3%	40	3%	41	2%
**Total**	**1,053**	**100%**	**1,494**	**100%**	**1,230**	**100%**	**1,696**	**100%**

**Table 4:** IPTc total financial and economic costs comparing OPD and EPI delivery strategies.

Delivery strategies	OPD delivery				EPI outreach delivery			
Number of children enrolled	248				244			
Costs	Total Financial (US$)	Cost Profile (%)	Total Economic (US$)	Cost Profile (%)	Total Financial (US$)	Cost Profile (%)	Total Economic (US$)	Cost Profile (%)
**IPTc drugs**	50	8%	50	6%	50	8%	50	6%
**Training**								
•Personnel	33	6%	78	9%	33	5%	78	9%
•Transport	5	1%	5	1%	5	1%	5	1%
**Delivering drugs to distribution points**								
•Personnel	15	3%	37	5%	15	2%	37	4%
•Transport	90	15%	83	10%	90	14%	83	10%
**Dispensing IPTc to parents/care-takers**								
•Personnel	240	40%	324	38%	240	38%	324	38%
•Transport	0	0%	0	0%	0	0%	0	0%
•Overheads	0	0%	30	3%	0	0%	23	3%
**Supervision of IPTc dispensing**								
•Personnel	40	7%	117	14%	51	8%	110	13%
•Transport	105	17%	99	12%	128	20%	123	14%
**Communications**	20	3%	20	2%	20	4%	20	2%
**Total**	**600**	**100%**	**843**	**100%**	**634**	**100%**	**853**	**100%**

IPTc was financially and economically less costly when dispensed by VHWs than by OPD or EPI nurses. The main financial cost drivers under the community-based delivery strategy were resources for delivering drugs to the dispensing sites (28%) and for supervision (28%) (Table 3). In comparison, under the facility-based strategy, these resources represented 18% and 26% of the total financial cost respectively (Table 3). These differences reflected the more remote location of villages that received IPTc through VHWs compared to those where treatment was delivered through health facilities; 190 kilometres were travelled each month to distribute drugs and conduct supervision under the VHW delivery strategy compared to 137 kilometres under the facility-based delivery approach. The main financial cost drivers under the facility-based delivery were resources for dispensing IPTc to parents/care takers (39%), reflecting the US$10.00 monthly incentive paid to 12 nurses working at OPD and EPI clinics compared to six VHWs dispensing IPTc in the community.

The main economic cost driver when IPTc was dispensed by VHWs was supervision, accounting for 32% of the total economic cost (Table 3). In comparison, when drugs were dispensed by facility-based nurses, supervision resources were responsible for 27% of the total economic cost (Table 3). This difference did not reflect more intense supervision of VHWs compared to nurses because supervisors spent around 40 minutes at each dispensing site per month. Instead, it was associated with longer distances travelled and, therefore, time spent by supervisors visiting VHWs compared to OPD and EPI nurses. Similarly, the relative importance of supervision costs was slightly higher when IPTc was dispensed by EPI rather than by OPD nurses (Table 4), reflecting the “outreach” nature of EPI dispensing sites. When IPTc was provided by facility-based nurses, the main economic cost driver was resources used for dispensing drugs (41%) (Table 3). This was higher than under the VHWs delivery strategy (26%) (Table 3). This reflected the time spent by the 12 nurses involved in dispensing IPTc (one-fifth of their working day during their respective IPTc days) that was valued using the gross monthly earning of a senior midwife amounting to US$367.00 per month whilst the time spent by six VHWs (three full days) was valued on the basis of a minimum monthly subsistence wage of US$34.00.

### Unit economic costs of IPTc

Table 5 shows the numbers and proportions of children fully covered, acceptably covered, fully covered and fully adherent, and acceptably covered and fully adherent under the different delivery strategies, and the corresponding unit economic cost per child. Detailed unit economic costs per child fully covered are presented in Table 6. The economic cost per child “fully covered” was US$4.58 when IPTc was delivered by VHWs and US$5.27 when delivered by nurses, resulting in an incremental saving of US$0.69 (Table 6). This difference in unit cost reflects lower total economic costs and higher coverage rates achieved under the community-based delivery strategy compared to the facility-based approach. IPTc delivered through VHWs was associated with the largest incremental saving when compared with the EPI outreach strategy (US$1.07 per child covered) (Table 6). For children who were “fully adherent”, VHW delivery was both more effective and less costly than the facility-based strategy: greater proportions of children took all three doses of four courses or all three doses of at least three courses using the community-based delivery strategy (Table 5), with incremental savings of US$0.95 per child “fully covered and fully adherent” and US$0.54 per child “acceptably covered and fully adherent”.

**Table 5:** IPTc unit economic costs for different outcome measures by delivery strategy.

	Number of children (%)				Economic unit cost per child (US$)			
	Community-based delivery (VHWs)	Facility- based delivery (OPD+EPI)	OPD delivery (OPD)	EPI outreach delivery (EPI)	Community-based delivery (VHWs)	Facility- based delivery (OPD+EPI)	OPD delivery (OPD)	EPI outreach delivery (EPI)
Children enrolled	472 (100%)	492 (100%)	248 (100%)	244 (100%)	$3.17	$3.45	$3.40	$3.50
Children who received the first supervised dose of 4 courses (children “fully” covered)	326 (69.1%)	322 (65.4%)	171 (69.0%)	151 (61.9%)	$4.58	$5.27	$4.93	$5.65
Children who received the first supervised dose of at least 3 courses (children “acceptably covered”)	442 (93.6%)	437 (88.9%)	228 (93.4%)	209 (84.3%)	$3.38	$3.88	$3.70	$4.08
Children who took all 3 doses of 4 courses (children “fully covered and fully adherent”)	210 (44.6%)	199 (40.5%)	n/a	n/a	$7.56	$8.51	n/a	n/a
Children who took all 3 doses of at least 3 courses (children “acceptably covered and fully adherent”)	305 (64.5%)	312 (63.5%)	n/a	n/a	$4.90	$5.44	n/a	n/a

n/a = data not available.

**Table 6:** IPTc economic costs per child receiving the first supervised dose of all four courses by delivery strategy.

Delivery strategies	Community-based delivery (VHWs)		Facility- based delivery (OPD+EPI)		OPD delivery (OPD)		EPI outreach delivery (EPI)	
Number of children who received at least the first dose of all 4 courses	326		322		171		151	
Costs	Unit Cost (US$)	Cost Profile (%)	Unit Cost (US$)	Cost Profile (%)	Unit Cost (US$)	Cost Profile (%)	Unit Cost (US$)	Cost Profile (%)
**IPTc Drugs**	0.29	6%	0.31	6%	0.29	6%	0.33	6%
**Training**								
•Personnel	0.45	10%	0.48	9%	0.45	9%	0.51	9%
•Transport	0.04	1%	0.03	1%	0.03	1%	0.04	1%
**Delivering drugs to distribution points**								
•Personnel	0.20	4%	0.23	4%	0.22	4%	0.25	4%
•Transport	0.81	18%	0.51	10%	0.48	10%	0.55	10%
**Dispensing IPTc to parents/care-takers**								
•Personnel	1.12	24%	2.02	38%	1.90	39%	2.15	38%
•Transport	0.00	0%	0.00	0%	0.00	0%	0.00	0%
•Overheads	0.09	2%	0.16	3%	0.17	4%	0.15	3%
**Supervision of IPTc dispensing**								
•Personnel	0.83	18%	0.70	14%	0.68	13%	0.73	13%
•Transport	0.62	14%	0.69	13%	0.58	12%	0.81	14%
**Communications**	0.13	3%	0.13	2%	0.12	2%	0.14	2%
**Total Unit Cost:**	**4.58**	**100%**	**5.27**	**100%**	**4.93**	**100%**	**5.65**	**100%**
**Incremental Saving (VHWs vs. FB)**	**0.69**							
**Incremental Saving (VHWs vs. OPD)**	**0.35**							
**Incremental Saving (VHWs vs. EPI)**	**1.07**							
**Incremental Saving (EPI vs.OPD)**	**−0.72**							

### Sensitivity Analysis

A series of univariate sensitivity analyses were conducted to test the impact of varying the costs of uncertain resources on IPTc unit economic costs. The sensitivity analyses aimed to provide a more realistic picture of IPTc economic outcomes outside the well supported and funded context of a trial. Results are presented in Table 7. IPTc dispensed by VHWs remained the least costly strategy under all scenarios, expect two. When the monthly incentives paid to VHWs were lowered from US$10.00 to US$5.00 and set to zero for nurses, it became less costly to deliver IPTc through the facility-based strategy, with a unit economic cost differential ranging across effectiveness outcomes between US$0.28 to US$0.80. When VHWs were trained for 5 days whilst nurses were assumed to receive IPTc training through the routine government curriculum, the unit cost differential ranged between US$1.37 toUS$2.58 in favour of the facility-based strategy.

**Table 7:** Sensitivity analyses on IPTc economic unit costs (US$) for different coverage and adherence outcomes comparing community- and facility-based delivery strategies.

Variable	Variation tested	Unit economic cost (US$)								Rationale for variation tested
		Child fully covered		Child acceptably covered		Child fully adherent & fully covered		Child fully adherent & acceptably covered		
		VHWs*	FB*	VHWs*	FB	VHWs*	FB	VHWs*	FB	
**Base case**	-	4.58	5.27	3.38	3.88	7.56	8.51	4.90	5.44	-
**Drug costs**	+25%	4.66	5.35	3.44	3.94	7.79	8.64	4.98	5.52	Uncertainty regarding cost of drugs in the future [24].
	−25%	4.51	5.19	3.33	3.82	7.33	8.38	4.82	5.36	
**Discount rate**	Increase from 3% to 5%	4.56	5.26	3.36	3.87	7.52	8.49	4.87	5.43	Alternative rate used in economic costing studies.
**Share of time spent on IPTc**	Nurses time increase from 20% to 30%	4.58	5.53	3.38	4.08	7.56	8.93	4.90	5.71	No evidence available on exact share of time dispensers spend on IPT activities in relation to their other tasks.
	VHWs time decrease from 100% to 50%	4.39	5.27	3.24	3.88	7.26	8.51	4.70	5.44	
**Incentives**	Decrease from US$10.00 to US$5.00 for VHWs and set to zero for nurses	4.09	3.72	3.02	2.74	6.80	6.00	4.37	3.84	In Ghana, community-based volunteers received US$8.00 quarterly (US$2.00 per month in addition to non-monetary benefits (raincoats, bicycles, etc) [22], or US$5.00 [23]
**Training intensity**	Increase from 1 day to 5 days for VHWs; nurses assumed to be trained on IPTc during routine government curriculum	6.45	4.59	4.75	3.38	10.00	7.41	6.89	4.74	In The Gambia, community-based volunteers were trained on IPTc for 5 days [6].

*VHWs = Village Health Workers; FB = Facility-Based delivery (OPD+EPI nurses).

## Discussion

The economic outcomes of this study are in agreement with the findings of a larger trial that investigated the cost-effectiveness of delivering IPTc (using SP+AQ) through the alternative strategies of VHWs and RCH trekking teams in The Gambia: VHWs proved to be a more cost-effective delivery strategy than the routine public health services. However, in Jasikan, the unit costs of delivering at least the first dose of all courses (US$4.58 when delivered by VHWs, US$4.93 by OPD nurses and US$5.65 by EPI nurses) were higher than in the Gambian study, during which the cost of delivering the first dose of all three courses was US$1.63 per child when dispensed by VHWs and US$3.47 when dispensed by RCH trekking teams [6]. However, in The Gambia, the intermittent treatment regimen was different than in our study as it included three courses of IPT compared to four, which brought down coverage cost. In addition, these cost differentials highlight the importance of the scale of IPTc delivery. With over 12,000 children enrolled, the Gambian trial benefited from economies of scale as fixed and semi fixed costs (training, incentives, and supervision) were spread over a larger number of children than in the Jasikan intervention, which included less than 1,000 children.

OPD and EPI delivery strategies led to lower coverage rates and therefore higher unit costs compared to delivery by VHWs, perhaps due in part to the fact that parents/care takers could collect their drugs over a two-day period at OPD and on only one day at EPI clinics compared to the three days on which drugs were available from VHWs.

During this study, there were uncertainties around the time spent by nurses on IPTc and the impact that this had on their routine tasks. In the selected OPDs, the workload of the nursing staff was manageable, so that no major impact on the quality of routine services was expected. At EPI outreach clinics, each nurse was estimated to serve annually an average of 1104 children aged between 0 to 4 years, equivalent to 92 children at each monthly clinic. Adding IPTc to their routine task inevitably increased nurses' workload and could lead to potential fatigue and, in the long-run, to negative effects on the overall quality of EPI services and IPTc. However, it is also important to consider that IPTc dispensed by health care professionals may create opportunities for children, such as the diagnosis of other diseases. Some of these issues have been explored in relation to IPTi delivery [9], [10] and are currently being debated in relation to the use of IPT in pregnant women. In the Gambia, four additional people were needed to support the delivery of IPTc by RCH trekking teams. Additional health personnel may, therefore, be required if IPTc is introduced as a malaria control intervention delivered through routine health services, notably those provided on an intermittent basis such as EPI and RCH programmes.

Whilst the EPI delivery strategy may have reached children from more remote areas than could be reached through OPD, EPI coverage in Jasikan is high and relatively equitable [11]. There are, therefore, additional uncertainties regarding the effectiveness of delivering IPTc through the EPI route in settings where EPI coverage is lower and/or inequitable, such as in several other countries of West Africa with seasonal malaria transmission. More generally, in many low income countries, public health services are disproportionately used by wealthier populations, who may be at lower risk of malaria and, if expected to live in more accessible areas, may benefit from other malaria control interventions. Community-based delivery strategies for ITNs have been reported to achieve higher coverage in lower socio-economic households than health facility-based alternatives [12] and such focussed delivery during a fixed time-period fits well with the delivery requirements of IPTc in areas with seasonal transmission of malaria.

Overall, impressive adherence rates were achieved with all three delivery strategies, although with higher rates achieved by VHWs than by facility-based nurses. IPT drugs were administered to children in sugared water and this approach may partly explain the high adherence levels achieved during this trial. Higher coverage and adherence achieved using the VHW strategy may have reflected preferences for patients/care givers to visit a member of their community that they know rather than a facility-based health worker. Visiting the former may also be more convenient as they can theoretically be visited at any time of the day as opposed to the limited opening hours of OPDs and EPI clinics. Parents/care-takers may also prefer services they can access locally and for which limited travel is required, as they commonly do when seeking fever malaria treatment from local drug shops [13], [14], [15], [16], [17], [18], [19]. Combining IPTc delivery with other malaria interventions, such as diagnosis using rapid test kits and treatment with artemisinin combination therapy, could be explored to decrease implementation costs. To ensure that they gave the right drug in the right dosage at the right time, VHWs would need support, notably in terms of training and supervision. In Jasikan, the trial benefited from good supervision from the DHMT, with monthly incentives of US$50.00 given to IPTc supervisors. In comparison, in The Gambia, supervisors did not receive any salary supplement. Instead, considerable efforts were made to support dispensers through job aids such as coloured cards. In addition, similar strategies could well be developed for parents/care takers. Finally, in Jasikan, dispensers received a monthly incentive of $10.00, which was similar to that received by VHWs in the neighbouring district of Hohoe but higher than the incentives given every quarter to VHWs involved in home based management of malaria [20], [21], [22] or in newborn care interventions [23]. Careful examinations of strategies and costs of strategies for sustaining networks of community-based volunteers need to be conducted. In conclusion, this study has shown that whilst high levels of coverage with IPTc can be achieved in a rural area of Ghana using delivery by VHWs or nurses working at OPDs or EPI outreach clinics, delivery by VHWs was less costly and had other non-monetary benefits.
